# Multicenter European Data of Anatomical Parameters of the Anterior Eye Segment Measured with an Optical Biometer

**DOI:** 10.3390/jcm14228192

**Published:** 2025-11-19

**Authors:** Ava Niknahad, Hyeck-Soo Son, Gerd U. Auffarth, Louise Blöck, Giacomo Savini, Catarina P. Coutinho, Joaquín Fernández, Grzegorz Łabuz

**Affiliations:** 1 The David J. Apple Center for Vision Research, Department of Ophthalmology, University of Heidelberg, 69120 Heidelberg, Germany; aniknah1@jhmi.edu (A.N.); hyecksoo.son@med.uni-heidelberg.de (H.-S.S.); louise.bloeck@med.uni-heidelberg.de (L.B.); grzegorz.labuz@med.uni-heidelberg.de (G.Ł.); 2 Johns Hopkins School of Medicine, Baltimore, MD 21205, USA; 3 IRCCS-G.B. Bietti Foundation, 00184 Rome, Italy; giacomo.savini@startmail.com; 4 Studio Oculistico d’Azeglio, 40123 Bologna, Italy; catarina.praefke@gmail.com; 5 Department of Pharmacy and Biotechnology, University of Bologna, 40126 Bologna, Italy; 6 Department of Ophthalmology, Qvision VITHAS Almería Hospital, 04120 Almería, Spain; joaquinfernandezoft@qvision.es

**Keywords:** normative, ocular data, Pentacam, aging, refractive error

## Abstract

**Background/Objectives**: Normative values of ocular parameters can be influenced by a variety of factors. This study evaluates the relationship of axial length (AL), anterior and posterior corneal curvature, anterior chamber, and corneal thickness variables with age, spherical equivalent (SE), and gender. **Methods**: A retrospective cohort study at three hospitals identified patients from June 2019 to July 2022. Eyes with no prior history of surgery and anterior segment pathology were included, with one eye per patient being examined by Pentacam AXL Wave (Oculus, Germany). **Results**: The 1075 patients included had a mean age of 52.9 (± 19.5 years), with 41.5% (446) identified as males. Compared to all variables, anterior chamber angle was most strongly associated with age, with a Spearman’s correlation coefficient (r) of −0.62 (*p* < 0.001), while AL was most strongly correlated with SE (r = −0.75, *p* < 0.001). The mean radius of anterior corneal curvature showed a significant positive correlation with SE (r = 0.08, *p* = 0.013). Between the two genders, males had larger median anterior chamber volume (157.8 versus 147.9 mm^3^, *p* < 0.001), depth (*p* < 0.05 internal and external), AL (24.2 versus 23.7 mm, *p* < 0.001), and flatter corneas (*p* < 0.05 anteriorly and posteriorly) compared to females. **Conclusions**: Age and SE were significantly negatively correlated with AL and anterior chamber parameters. While males showed longer AL and deeper and larger anterior chambers, females had steeper corneas but similar anterior chamber angles. The differences warrant considering age, SE, and gender when interpreting a patient’s examinations against normative data.

## 1. Introduction

Obtaining normative values of ocular parameters has been paramount in diagnosing and monitoring ocular diseases. While this task appears simple enough, it has been shown that the interpretation of the obtained normative data is challenging, as the results are influenced by a multitude of factors, such as the machine used to obtain the variables, gender, age, and refractive error. For example, axial length (AL) measured by IOL Master (Carl Zeiss Meditec AG, Jena, Germany) has been shown to be reported significantly shorter than the same patient’s AL when measured by Pentacam AXL (Oculus Optikgeräte GmbH, Wetzlar, Germany), but to be similar to the results measured by ANTERION SS-OCT (swept source optical coherence tomography) (Heidelberg Engineering, Heidelberg, Germany) [1]. AL has additionally been reported to be associated with age in a mixed way in the literature. While Fotedar et al. found a decrease in AL of 0.09 mm per decade of life, Atchison et al. reported an increase of 0.011 mm per year of life [2,3]. AL has been found to be longer in males than in females [4,5] and to be significantly negatively correlated with refraction [4,6].

Like AL, other variables of the eye’s anterior segment have been shown to be similarly influenced, but the current studies are limited by each evaluating a different parameter and exploring only some of the related dependent variables [7,8]. This study aims to address this gap by evaluating a series of anterior segment variables across a large cohort of patients imaged by Pentacam AXL Wave, and it reports their association with age, gender, and refractive errors. Pentacam was the device chosen for imaging patients, as it is one of the most commonly used devices by ophthalmologists for detecting and monitoring keratoconus [9] and preoperative screening for refractive and cataract surgery [10,11], with the Pentacam AXL Wave carrying additional features such as ectasia detection, objective refraction, visual performance analysis for patient education, and intraocular lens calculation for various corneal shapes [12]. By providing normative ocular data using Pentacam AXL Wave, we hope to provide a comprehensive report of AL, anterior and posterior corneal curvature, corneal thickness, and anterior chamber variables that can be adjusted according to patients’ age, gender, and refractive error in cases where patient characteristics impact their results in a significant way.

## 2. Materials and Methods

This retrospective cohort study was conducted at the University Eye Hospital of Heidelberg, Germany; the Department of Ophthalmology (Qvision) at the Vithas Hospital in Almería, Spain; and G.B. Bietti in Rome, Italy, from June 2019 to July 2022. Research was conducted in compliance with the human ethics committee at each institution and followed the tenets of the Declaration of Helsinki. The Institutional Review Board approval numbers were 24/15/FB for IRCCS Bietti Foundation (approved in 2015), 202499908562931 for Qvision at the Vithas Hospital (approved in 2024), and S-392/2011 for the University Eye Hospital of Heidelberg (approved in 2019). Eyes were excluded from this study if they (1) had a history of ocular surgeries, including cataract or refractive surgery, or (2) demonstrated anterior segment pathologies such as keratoconus, Fuchs corneal endothelial dystrophy, corneal scarring, or ocular trauma (based on either slit-lamp examination or Scheimpflug imaging). For the eyes that met the inclusion criteria, the recorded examinations were performed at the presenting clinics before performing other examinations or applying any eye drops. Given that only one eye per patient was used for analysis, for patients for whom both eyes met the inclusion criteria, an eye was chosen at random for inclusion in the analysis.

Anterior eye segment parameters were acquired from Pentacam AXL Wave (OCULUS Optikgeräte GmbH, Wetzlar, Germany)—a device that combines Scheimpflug-based tomography, optical biometry (partial coherence interferometry—PCI), and Hartmann–Shack aberrometry and accomplishes five purposes: tomography of the anterior eye segment, AL measurement, objective refraction, wavefront aberrometry of the entire eye, and retro-illumination. Exclusively, measurements with a good quality score (QS = OK) were included in the analysis. Parameters of interest included AL, anterior and posterior corneal curvature, corneal thickness, and anterior chamber variables. Corneal curvature variables included posterior to anterior corneal curvature ratio (BF ratio), anterior and posterior corneal asphericity (Q (front) and Q (back)), and mean radius of anterior and posterior corneal curvature (Rmean (front) and Rmean (back)). Corneal thickness variables included corneal thickness at the apex (PachyApex), minimum corneal thickness (PachyMin), and their difference (PachyDiff). Anterior chamber variables included horizontal white-to-white (HWTW), anterior chamber angle (ACA), anterior chamber volume (ACV), and anterior chamber depth (ACD), both internal (ACDint) and external (ACDext), defined as measurements from the endothelium and epithelium, respectively. In addition to the mentioned eye examination variables, patients’ age, gender, and spherical equivalent (SE) measured in diopters (D) were recorded.

Statistical analyses and data presentation were conducted using Statistics and Machine Learning Toolbox (MATLAB, R2024b, MathWorks, Natick, MA, USA). The data were reported as the mean, standard deviation (SD), median, minimum, maximum, and 95% confidence interval (CI). The normality of the data distribution was determined by the Kolmogorov–Smirnov test. Since not all variables were normally distributed, non-parametric tests, i.e., Mann–Whitney U-tests, Spearman, and Quantile Regression Analysis, were applied for statistical comparisons.

## 3. Results

In total, 1075 patients, including 446 (41.5%) males and 629 (58.5%) females, with a mean age of 52.9 ± 19.5 years, met the inclusion criteria. Of the 1075 eyes included, 454 (42.2%) were right and 621 (57.8%) were left eyes. A descriptive summary of the studied parameters is included in Table 1. Of all the variables, age, SE, ACA, ACV, PachyDiff, HWTW, AL, Q (front), and Q (back) were normally distributed. Overall, patients were found to have a mean SE of −1.81 D (±3.76 D), ACDint of 2.86 mm (±0.43 mm), ACDext of 3.40 mm (±0.43 mm), PachyApex of 541.3 µm (±34.0 µm), AL of 24.1 mm (±1.5 mm), and BF ratio of 81.94% (±1.60%).

**Figure 1:**
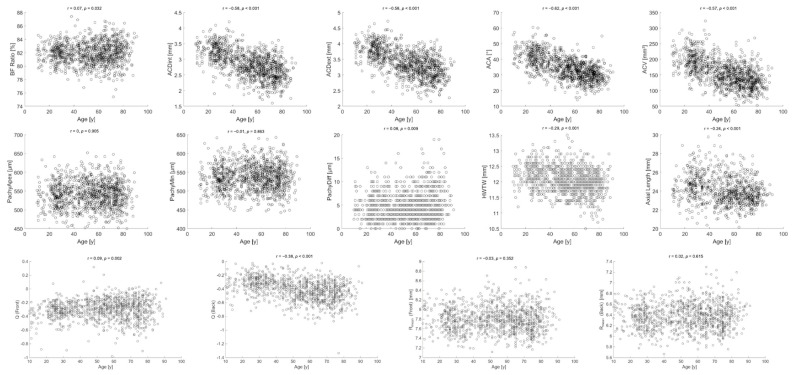
Variations in all anterior segment parameters across age. r: Spearman’s correlation coefficient; y: years; BF Ratio: ratio of posterior to anterior corneal curvature; ACDint: anterior chamber depth—internal; ACDext: anterior chamber depth—external; ACA: anterior chamber angle; ACV: anterior chamber volume; PachyApex: corneal thickness at the apex; PachyMin: minimum corneal thickness; PachyDiff: difference between PachyApex and PachyMin; HWHW: horizontal white-to-white; Q: corneal asphericity; Rmean: mean radius of corneal curvature.

The variations in all anterior segment parameters across ages are displayed in Figure 1. All examined variables except PachyApex, PachyMin, Rmean (front), and Rmean (back) were found to be statistically correlated with age. While the BF ratio and PachyDiff were positively correlated with age (r coefficients of 0.07 and 0.009, respectively), PachyApex was found not to be correlated with age in any direction (r = 0), while other variables were negatively correlated with age. It is notable to mention that such r correlations for the B ratio and PachyDiff, however, are likely clinically insignificant. ACA was most strongly negatively correlated with age at Spearman’s correlation coefficient of −0.62 (*p* < 0.001).

**Figure 2:**
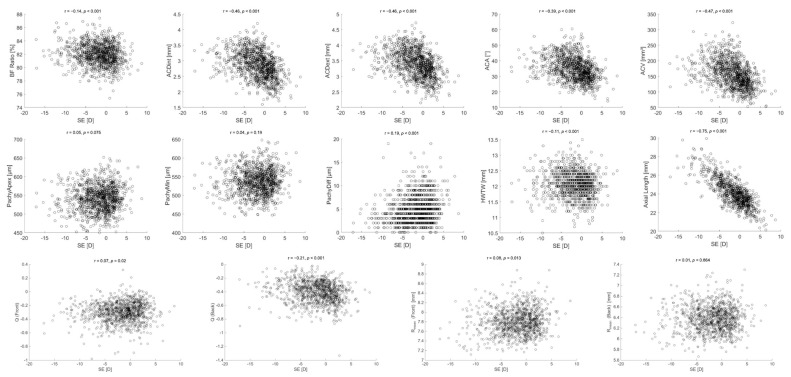
Variations in all anterior segment parameters across refractive error. SE: spherical equivalent; D: diopters; r: Spearman’s correlation coefficient; BF Ratio: ratio of posterior to anterior corneal curvature; ACDint: anterior chamber depth—internal; ACDext: anterior chamber depth—external; ACA: anterior chamber angle; ACV: anterior chamber volume; PachyApex: corneal thickness at the apex; PachyMin: minimum corneal thickness; PachyDiff: difference between PachyApex and PachyMin; HWTW: horizontal white-to-white; Q: corneal asphericity; Rmean: mean radius of corneal curvature.

Figure 2 displays the variations in all anterior segment parameters across SE (D). All variables except PachyApex, PachyMin, and Rmean (Back) were statistically significantly correlated with SE, and all variables except PachyApex, PachyMin, PachyDiff, and Q (back) were negatively correlated with increasing SE. Increasing axial length showed the strongest negative correlation with increasing SE (r = −0.75, *p* < 0.001), while Rmean (front) showed a small statistically significant positive, however likely clinically insignificant correlation with r = 0.08 (*p* = 0.013), and Rmean (back) showed no significant correlation (r = 0.01, *p* = 0.864).

A descriptive summary of all variables compared between the two genders is displayed in Table 2. Notably, males had a significantly higher median age at the time of examination compared to females (median 60.0 versus 53.0 years old, *p* < 0.001). While ACA and ACD parameters were similar between the two genders, ACV was significantly larger in males compared to females (median 157.80 versus 147.90 mm^3^, *p* < 0.001). Median PachyDiff was significantly higher at 5.0 versus 4.0 µm for male versus female patients (*p* = 0.006). Male patients additionally had longer AL compared to female patients (median 24.22 versus 23.65 mm, *p* < 0.001). Female patients had significantly steeper anterior and posterior corneal curvatures (both *p* < 0.001), while anterior and posterior corneal asphericity were similar between the two genders.

**Figure 3:**
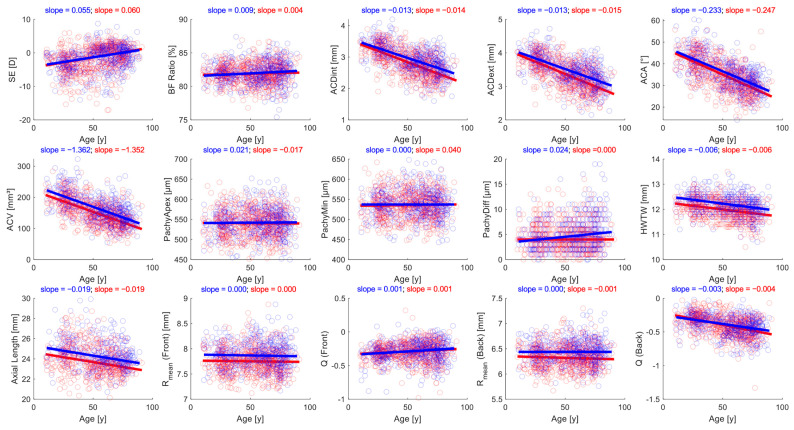
Variations in all anterior segment parameters across age by gender: female (red), male (blue). SE: spherical equivalent; D: diopters; y: years; BF Ratio: ratio of posterior to anterior corneal curvature; ACDint: anterior chamber depth—internal; ACDext: anterior chamber depth—external; ACA: anterior chamber angle; ACV: anterior chamber volume; PachyApex: corneal thickness at the apex; PachyMin: minimum corneal thickness; PachyDiff: difference between PachyApex and PachyMin; HWTW: horizontal white-to-white; Rmean: mean radius of corneal curvature; Q: corneal asphericity.

Similar to Figure 1, Figure 3 shows the variation in the parameters by age, but separately for male and female patients. The two genders showed different associations with age, especially regarding PachyApex, where males were positively associated with increasing age, while females were negatively associated with increasing age (slope of the best-fit line, respectively, 0.021 and −0.017). While males showed no association with increasing age for PachyMin and females for PachyDiff, females showed a positive association for PachyMin with increasing age (slope = 0.040), and males showed a positive association for PachyDiff with increasing age (slope = 0.024).

## 4. Discussion

This retrospective study reported the normative values of AL, anterior chamber, corneal thickness, and curvature variables in 1075 eyes across three university hospital centers using Pentacam AXL Wave. While the majority of the mean values were included in the range reported in the literature, the ranges and discrepancies themselves emphasize the value of obtaining normative data across various biometers. Anterior chamber parameters and AL were significantly negatively correlated with both age and SE. PachyApex and PachyMin did not demonstrate any correlation. Males were found to have longer AL and larger ACD and ACV than females. While the ACA values between the two genders were similar, females were found to have a faster decrease in ACA over an increase in age at −0.247°/year compared to −0.233°/year in men. The highlighted impacts of the ocular parameters by the discussed factors underline the importance of interpreting the normality of patients’ ocular findings on an individual basis.

In this study, the summary of the normative data for anterior segment of eye parameters in the majority agreed with the current reported literature. In our cohort of 1075 patients, the mean AL of 23.94 mm was consistent with the range of mean AL values of 23.23 to 25.17 mm reported in the literature when measured using IOL Master and A-scan ultrasound [2,6,13,14,15]. Similarly, our reported mean BF ratio of 81.9% was included in the mean BF ratio range of 81.37% to 84.03% when measured by Scheimpflug camera, optical coherence tomography, and slit-scan topography [13,16,17,18,19]. Our mean corneal thickness parameters were included in the mean range reported in the literature, with a mean PachyMin of 536 µm in the 526.92 to 552.56 µm range, a mean PachyApex of 541 µm in the 530.06 to 558.45 µm range, and a mean PachyDiff of 4 µm in the 3.14 to 7 µm range [7,20,21]. This consistency suggests that the normative data reported across such studies are reproducible and reliable, with our study’s patients being an appropriate sample of the population for whom normative data have been reported.

When measured via Pentacam, previously reported mean HWTW ranged from 11.75 to 12.07 mm, which includes our mean HWTW value of 12.06 mm [15,22,23]. The mean ACA in the literature ranges from 32.6 to 37.12°, which is inclusive of our mean value of 34.3° [8,24,25]. However, our mean ACV, ACDint, and ACDext values were outside of the reported mean ACV and ACD ranges. This study’s mean ACV of 154.2 mm^3^ was lower than the mean ACV range of 160.3 to 207.93 mm^3^ [8,24,26]. Our mean ACDint of 2.85 mm and ACDext of 3.40 mm were within the mean ACD range of 2.71 to 3.41 mm found in the literature when measured by Pentacam and IOL Master [1,2,6,8,15,24,25,26]. It is notable to mention that both Taña-Rivero et al. and Song et al. have shown that the ACD values of the same subjects can be significantly different between various devices. For example, Taña-Rivero et al. found significant differences between ACD between Pentacam AXL PCI, IOL Master 700 SS-OCT, and ANTERION SS-OCT [1], while Song et al. found significant differences between ACD between Pentacam, Sirius (CSO, Italy), and IOL Master 700 [27]. Various biometers measure ACD differently and may report ACD only as external or internal, such as the IOL Master, which only measures external ACD. To help compare the normative ACD values of future studies to the current literature, it may be best to report ACD specifically as ACDint or ACDext and to continue emphasizing the device used to collect these values. For patients, their ACD values may be monitored and compared to normative data with more ease if a consistent biometer is used for their evaluations for the duration of their follow-up.

Anterior chamber variables have been shown to significantly negatively correlate with age [3,5,6,28], a change thought to result from age-related lens thickening [29,30]. In our study, ACDint, ACDext, ACV, and ACA similarly had the highest negative Spearman correlation coefficient with aging, with a minimum coefficient of −0.57 for ACV. In agreement with our findings, in a study of 666 subjects aged 3 to 85 years old, Orucoglu et al. also found high Pearson correlation coefficients for age with these variables, with −0.512 for ACV, −0.481 for ACD, and −0.408 for ACA (all *p*-values < 0.001), respectively [8]. HWTW was also found to be negatively significantly correlated with age, consistent with a cross-sectional study of 1721 patients by Fu et al.; however, it had a lower Spearman correlation coefficient of −0.24 compared to the other mentioned anterior chamber variables [23].

We found PachyMin and PachyApex to not be significantly correlated with age, with both showing weak Spearman correlation coefficients (r = −0.01, *p* = 0.863 and r = 0, *p* = 0.905). Similarly, in a study of 95 myopic eyes, Roshdy et al. found that mean PachyApex and PachyMin did not significantly differ among patients younger than 21 years old, those between 21 and 40 years old, and those older than 40 years old [31]. However, Vitályos et al., in a prospective study of 35 eyes, reported a mean PachyMin decrease from 548 to 534 µm over 3.6 years (*p* < 0.001) [32]. While the literature on PachyMin and PachyApex correlation with age is scarce, the literature on the CCT agrees with our findings on the PachyApex and PachyMin relationship with age. While age is related to CCT [33,34], not all studies have found significant correlations [35]. CCT has instead shown significant changes with a variety of other factors, such as consistent, strong correlations with ethnicity [33]. Therefore, our study may support the lack of a significant correlation of PachyMin and PachyApex with age, suggesting the corneal thickness does not significantly change throughout one’s life in non-pathologic eyes. Although given the opposing literature on this topic, future studies can help better reveal if such a correlation exists by examining corneal thickness parameters across a larger variety of factors.

We found spherical equivalent uncorrelated with either PachyApex or PachyMin, similar to other studies in the literature [36,37,38], although hyperopes and myopes appeared to differ in respect to AL, agreeing with the literature that suggests ocular shape changes associated with SE changes. AL has been reported in multiple studies to decrease with an increase in refractive error [6,39]. In a study of 723 eyes in patients aged 55 years and older, AL measured using ultrasound biometry was found to be significantly negatively correlated with SE, with an r of −0.59 (*p* < 0.0001) [4]. Similarly, our study found this correlation to be −0.75 with *p* < 0.001. Compared to AL findings, in our study, corneal curvature findings such as Rmean (front) only had a small positive correlation with SE (r = 0.08, *p* = 0.013), and Rmean (back) was not significantly correlated with SE (*p* = 0.864). In a study of 500 patients aged 40–80 years, Chen et al. similarly reported a stronger correlation between SE and AL with r = −0.645 (*p* < 0.001) compared to the correlation between SE and corneal curvature (r = 0.013, *p* = 0.770) [40]. Refractive error thus appears to have a stronger association with AL compared to corneal curvature.

In addition to shorter AL, patients with hyperopia are known to have smaller ACD, ACA, and ACV [8,28]. We found the Spearman’s correlation coefficient between SE and ACD, ACV, and ACA to be −0.46, −0.47, and −0.39, similar to those reported by Orucoglu et al. at −0.475, −0.504, and −0.414, respectively [8]. He et al. reported an ACD decrease of 0.03 per +1D increase in SE, and Alsaif et al. reported a significant decrease in ACD (from 3.26 to 3.19 mm) and ACV (from 204.52 mm^3^ to 197.70 mm^3^) between moderately (−3 to −5.75 D) and severely (≥−6.00 D) myopic patients [28,37]. Overall, our study, along with the literature, collectively concludes that anterior chamber and AL variables are significantly impacted by the refractive error of the patients, and refractive error should be taken into consideration when interpreting reported normative values.

While our findings on gender differences on AL, ACD, ACV, and corneal thickness agree with the current literature, we found ACA and BF ratios to be similar between the two genders. We found AL to be 0.57 mm smaller for the median female AL compared to the median male AL (*p* < 0.001), which was in the range of the mean AL difference of 0.54 to 0.62 mm reported by previous studies in the literature [2,3,4]. Both genders in our report experienced a decrease in AL by age at a rate of −0.019 mm/year, most likely indicating a cohort effect, as in our cross-sectional studies, younger adults may have higher body length or higher prevalence of myopia compared to older adults [41]. However, further studies are needed to investigate the impact of age-related changes in ocular media refractive indices on AL measurements. While our anterior chamber parameters, ACD and ACV, were expectedly lower for women than men [5,8,28], we found ACA to be similar between the two genders (*p* = 0.144). For example, Orucoglu et al., in a study of 666 adults, reported a mean ACA increase of 1.97° in males compared to females (*p* = 0.002) [8]. When looking at the rate of ACA change across age between the two genders, females appeared to have a faster decrease in ACA per year at −0.247°/year compared to males at −0.233°/year. Given that our population included children in addition to adults compared to the Orucoglu et al. study of adult patients, the ACA changes between genders may be the result of ACA changes as children transition to adults. However, longitudinal prospective studies are needed to elucidate possible ACA changes over time across genders.

Lastly, females have been found to have steeper corneal curvature than males through parameters such as average corneal power [42,43], agreeing with our findings comparing the mean radius of anterior and posterior corneal curvature (both *p* < 0.001). To our knowledge, the gender differences in the BF ratio have not yet been explored. In our cohort, females had a similar BF ratio to males at 82.00% and 81.90% (*p* = 0.494), while both their Rmean (front) and Rmean (back) were lower than those of males (*p* < 0.001). It therefore appears that while females may have steeper corneal curvatures compared to males, the posterior-to-anterior curvature ratio between the two genders remains similar.

Overall, while our study provided a normative summary of various ocular parameters with their relationship to age, refractive error, and gender, it was limited in a few ways. As mentioned, the type of biometer used for measurements may lead to significantly different outcomes [44,45]; thus, our normative data may only be helpful in settings where a similar biometer to our study was used, as discrepancies between device findings may have clinical implications. For example, intraocular lens power calculations significantly rely on anterior segment measurements, and variations between devices may lead to different effective lens position predictions or IOL power recommendations [46]. Second, this study could not evaluate all the possible corneal parameters that one may be interested in. Additionally, the population of our study was limited to European patients. Ethnicity is a large contributor to different ocular parameters such as ACD and ACA [47,48]; thus, our normative values may not apply to all ethnicities. It is notable to mention that the mean value of all reported variables included the range of mean values reported by the literature across various ethnicities and biometers. Lastly, given the cross-sectional nature of this study, the reported associations between age and the eye segment variables may reflect cohort effects. For example, the prevalence of myopia has been increasing in younger generations [49], and the association between age and axial length may reflect such an increase. Future cohort studies, if longitudinally performed, may better control for effects that may impact the interpretation of age-related changes in anterior eye segment parameters.

## 5. Conclusions

In conclusion, this study provided normative data for anterior segment of eye parameters for 1075 patients (1075 eyes) with a mean age of 52.9 years measured by Pentacam AXL Wave. Although the tested corneal thickness parameters were not associated with age or SE, AL and anterior chamber parameters were significantly negatively associated with both age and SE. While males showed larger AL, ACD, and ACV than females, ACA and BF ratios were similar between the two cohorts, and corneal curvature values were lower for females compared to males. Future studies looking at similar parameters across age, refractive error, and gender of populations of different ethnicities using different biometers can help provide comprehensive normative data for a larger population.

## Figures and Tables

**Table 1:** Descriptive summary of anterior segment parameters in all patients. SD: standard deviation; D: diopter; BF Ratio: ratio of posterior to anterior corneal curvature; ACDint: anterior chamber depth—internal; ACDext: anterior chamber depth—external; ACA: anterior chamber angle; ACV: anterior chamber volume; PachyApex: corneal thickness at the apex; PachyMin: minimum corneal thickness; PachyDiff: difference between PachyApex and PachyMin; HWTW: horizontal white-to-white; Q: corneal asphericity; Rmean: mean radius of corneal curvature.

Variable	Median (Min, Max)	Mean ± SD
Age (years)	56.0 (10.0, 91.0)	52.9 ± 19.5
Spherical Equivalent (D)	−1.25 (−17.11, 8.72)	−1.81 ± 3.76
BF Ratio (%)	81.90 (75.40, 87.40)	81.94 ± 1.60
ACDint (mm)	2.85 (1.60, 4.20)	2.86 ± 0.43
ACDext (mm)	3.40 (2.15, 4.72)	3.40 ± 0.43
ACA (°)	34.3 (14.1, 60.5)	34.8 ± 7.4
ACV (mm^3^)	154.20 (52.70, 322.80)	156.82 ± 43.16
PachyApex (µm)	541.0 (450.0, 652.0)	541.3 ± 34.0
PachyMin (µm)	536.0 (443.0, 648.0)	536.5 ± 34.0
PachyDiff (µm)	4.0 (0.0, 19.0)	4.8 ± 2.9
Axial Length (mm)	23.94 (20.15, 29.93)	24.11 ± 1.47
HWTW (mm)	12.10 (10.50, 13.50)	12.06 ± 0.41
Q (front)	−0.29 (−0.98, 0.32)	−0.30 ± 0.15
Q (back)	−0.39 (−1.33, −0.02)	−0.41 ± 0.15
Rmean (front) (mm)	7.79 (7.12, 8.88)	7.80 ± 0.26
Rmean (back) (mm)	6.38 (5.67, 7.30)	6.38 ± 0.24

**Table 2:** Descriptive summary of anterior segment parameters by gender. CI: confidence interval; SE: spherical equivalent; D: diopter; BF Ratio: ratio of posterior to anterior corneal curvature; ACDint: anterior chamber depth—internal; ACDext: anterior chamber depth—external; ACA: anterior chamber angle; ACV: anterior chamber volume; PachyApex: corneal thickness at the apex; PachyMin: minimum corneal thickness; PachyDiff: difference between PachyApex and PachyMin; HWTW: horizontal white-to-white; Q: corneal asphericity; Rmean: mean radius of corneal curvature. * *p* < 0.05, ** *p* < 0.01, *** *p* < 0.001.

Parameter	Female Median [95% CI], Min, Max	Male Median [95% CI], Min, Max	*p*-Value
Age (years)	53.0 [16.0–81.8], Min = 10.0, Max = 91.0	60.0 [15.7–84.0], Min = 11.0, Max = 89.0	<0.001 ***
SE (D)	−1.32 [−11.25–4.01], Min = −17.11, Max = 6.38	−1.18 [−9.71–4.16], Min = −13.99, Max = 8.72	0.956
BF Ratio (%)	81.90 [78.70–84.78], Min = 76.80, Max = 86.90	82.00 [78.60–85.13], Min = 75.40, Max = 87.40	0.494
ACDint (mm)	2.83 [2.04–3.62], Min = 1.77, Max = 3.89	2.88 [1.98–3.72], Min = 1.60, Max = 4.20	0.026 *
ACDext (mm)	3.37 [2.60–4.17], Min = 2.29, Max = 4.41	3.43 [2.56–4.25], Min = 2.15, Max = 4.72	0.018 *
ACA (°)	34.1 [21.7–48.6], Min = 15.7, Max = 55.7	34.6 [22.5–51.0], Min = 14.1, Max = 60.5	0.144
ACV (mm^3^)	147.90 [82.22–238.38], Min = 52.70, Max = 283.40	157.80 [86.46–261.85], Min = 55.20, Max = 322.80	<0.001 ***
PachyApex (µm)	540.0 [477.0–605.8], Min = 452.0, Max = 642.0	542.0 [475.3–618.7], Min = 450.0, Max = 652.0	0.128
PachyMin (µm)	536.0 [473.2–602.8], Min = 447.0, Max = 642.0	537.0 [471.9–612.4], Min = 443.0, Max = 648.0	0.211
PachyDiff (µm)	4.0 [1.0–11.0], Min = 0.0, Max = 16.0	5.0 [1.0–12.0], Min = 0.0, Max = 19.0	0.006 **
Axial Length (mm)	23.65 [21.47–27.17], Min = 20.15, Max = 28.72	24.22 [21.96–27.72], Min = 20.71, Max = 29.93	<0.001 ***
HWTW (mm)	12.00 [11.26–12.70], Min = 10.50; Max = 13.50	12.20 [11.30–13.00] Min = 10.70; Max = 13.40	<0.001 ***
Q (front)	−0.30 [−0.59–−0.06], Min = −0.98, Max = 0.32	−0.28 [−0.64–−0.02], Min = −0.89, Max = 0.11	0.395
Q (back)	−0.39 [−0.71–−0.11], Min = −1.33, Max = −0.03	−0.39 [−0.76–−0.16]. Min = −0.87, Max = −0.02	0.347
Rmean (front) (mm)	7.75 [7.27–8.28], Min = 7.12, Max = 8.88	7.87 [7.43–8.40], Min = 7.17, Max = 8.88	<0.001 ***
Rmean (back) (mm)	6.32 [5.91–6.83], Min = 5.67, Max = 7.30	6.44 [6.00–6.90], Min = 5.76, Max = 7.24	<0.001 ***

## Data Availability

Data is available on reasonable request from the corresponding author and in compliance with all the study’s institutions’ Ethics Committee regulations.
